# An Integrated and Interdisciplinary Model for Predicting the Risk of Injury and Death in Future Earthquakes

**DOI:** 10.1371/journal.pone.0151111

**Published:** 2016-03-09

**Authors:** Stav Shapira, Lena Novack, Yaron Bar-Dayan, Limor Aharonson-Daniel

**Affiliations:** 1 PREPARED—Center for Emergency Response Research, Ben-Gurion University of the Negev, Beer-Sheva, Israel; 2 Department of Emergency Medicine, Leon and Mathilde Recanati School for Community Health Professions, Faculty of Health Sciences, Ben-Gurion University of the Negev, Beer-Sheva, Israel; 3 Department of Public Health, Faculty of Health Sciences, Ben-Gurion University of the Negev, Beer-Sheva, Israel; University of Vermont, UNITED STATES

## Abstract

**Background:**

A comprehensive technique for earthquake-related casualty estimation remains an unmet challenge. This study aims to integrate risk factors related to characteristics of the exposed population and to the built environment in order to improve communities’ preparedness and response capabilities and to mitigate future consequences.

**Methods:**

An innovative model was formulated based on a widely used loss estimation model (HAZUS) by integrating four human-related risk factors (age, gender, physical disability and socioeconomic status) that were identified through a systematic review and meta-analysis of epidemiological data. The common effect measures of these factors were calculated and entered to the existing model’s algorithm using logistic regression equations. Sensitivity analysis was performed by conducting a casualty estimation simulation in a high-vulnerability risk area in Israel.

**Results:**

the integrated model outcomes indicated an increase in the total number of casualties compared with the prediction of the traditional model; with regard to specific injury levels an increase was demonstrated in the number of expected fatalities and in the severely and moderately injured, and a decrease was noted in the lightly injured. Urban areas with higher populations at risk rates were found more vulnerable in this regard.

**Conclusion:**

The proposed model offers a novel approach that allows quantification of the combined impact of human-related and structural factors on the results of earthquake casualty modelling. Investing efforts in reducing human vulnerability and increasing resilience prior to an occurrence of an earthquake could lead to a possible decrease in the expected number of casualties.

## Introduction

Despite immense efforts invested in disaster risk reduction around the world, earthquakes continue to claim a heavy toll and remain the deadliest natural disaster worldwide, as demonstrated in recent events such as the 2010 Haiti and 2015 Nepal earthquakes [1–3].

Dense urban population centers are known as highly vulnerable in this context [4]. Nevertheless, evidence suggests that several specific population groups are disproportionately affected and face higher risk of earthquake-induced injury and death. Data gathered from epidemiological studies, demonstrates repeatedly that certain risk factors such as gender, age, and physical disability increase vulnerability to earthquakes’ adverse consequences. This increase is more dramatic in developing countries that lack the resources to support and augment disaster preparedness and response efforts, adding socioeconomic status as another risk factor in this regard [5–7]. The reasons for the increased vulnerability of these populations is not always clear. One possible explanation is the link between a potential decline in mobility and inability to adopt protective behaviors such as fleeing collapsing buildings during the tremor [5,6,8].

Future challenges in the field of disaster risk reduction thus require a more diverse, people-centered preventive approach. An efficient disaster risk management program should be founded on preliminary evaluation of vulnerabilities and potential risks to the population, structures and infrastructures; such knowledge can be leveraged for improving pre-disaster preparedness and mitigation and for the development of an effective response [9]. Loss-estimation models are considered an effective tool for assessing earthquake risks and potential consequences prior to occurrence. One internationally used model is HAZUS, developed in the US by the Federal Emergency Management Agency (FEMA) and the National Institute of Building Science (NIBS) [10–11]. Hazus produces quantitative estimations of earthquake losses, among them, the expected number of casualties. This model and other similar models, base their estimates on the association between damage to the built environment and the number of casualties [10,12–13] The probabilities of structures in the inspected area to be damaged in a given earthquake are estimated using engineering methods (appropriate fragility functions), and once this aspect is evaluated, the expected number of casualties associated with different building damage states (slight, moderate, extensive, and complete with or without collapse) is obtained by multiplying predefined casualty rates by the number of occupants presumably present in structures at the time of the event, according to local census data. Casualty rates are derived based on a combination of historical data and expert opinions [10,14] and are classified on a four level injury severity scale: 1) light, 2) moderate, 3) severe, and 4) fatal. As mentioned above, the model is engineering-based and does not take into consideration human-related factors such as population characteristics, a fact that may compromise its predictive accuracy.

Israel is situated along the Dead Sea Fault, which has been the origin of intensive earthquakes causing widespread devastation for over 2000 years. Although no major earthquake has struck the region in the last 90 years, experts forecast that strong tremors might occur in the near future and stress the need for action in the region, which is almost entirely located in a seismic risk zone [11].

The aim of this study is to produce an integrative and interdisciplinary model for estimating earthquake casualties in a high risk area in Israel, using risk factors associated with both the built environment and the population’s characteristics. The model structure is generic and can be applied in different regions depending on their specific population characteristics and available data.

## Materials and Methods

### Study design and conceptual approach

The new model was formulated based on the HAZUS casualty estimation model. Several human related factors that were found as increasing the risk of injury and death in earthquakes were integrated into the current HAZUS model; and a sensitivity analysis was performed in order to assess how the addition of these parameters affects the casualty projections.

### Model formulation

#### Human-related predictors and meta-analysis procedure

The predictors used in this model were identified based on a previous peer-reviewed systematic analysis aimed to assess individual and household characteristics associated with earthquake-induced death and injury in previous events; the review included studies with an analytical design that reported effect size measures, and the results revealed four risk-factors that increased human vulnerability to earthquakes. These were: gender (female); age (>65 years); having a physical disability; and belonging to a low socioeconomic status [5].

Eight studies were identified and included in a meta-analysis aimed to compute the combined effect for each risk-factor on the probability of an individual to (a) die, or (b) suffer an injury in an earthquake [15–22]. Information regarding the studies included in this analysis and effect sizes extracted from each study are detailed in Table 1. Since the studies estimated the effect size in different events and communities, which varied in their characteristics, a random effect model was fitted to calculate the combined effects [23]. For the purpose of the procedure, all effect size measures were converted to a unified format of odds ratio (OR) and 95% confidence intervals. The analysis was conducted using the ‘Comprehensive Meta-Analysis’ (CMA) software, version 3.

**Table 1:** Studies included in meta-analysis of human-related risk factors for injury and death in earthquakes.

Risk factor	Publication	Event(s)	Assessed risk of-	OR	95% CI	Combined effect
Age						
	Liang et al, 2001	1999 Chi-Chi, Taiwan	Death	1.1	1.0–1.1	2.9 (0.9–8.6)
	Chou et al, 2004	1999 Chi-Chi, Taiwan	Death	5.5	4.4–6.8	
	Dong et al, 2012	2012 Sichuan	Injury	4.6	1.8–11.5	1.3 (0.3–5.3)
	Doocy et al, 2013	2010 Haiti	Injury	2.8	1.6–4.7	
	Shoaf et al, 1998	Whittier Narrows 1987; Loma Prieta 1989; Northridge 1994; all in California, USA	Injury	0.7	0.5–0.8	
	Peek-Asa et al, 2003	1994 Northridge, California	Injury	2.9	1.2–7.4	
Gender						
	Chou et al, 2004	1999 Chi-Chi, Taiwan	Death	1.2	1.1–1.3	1.2 (1.1–1.3)
	Shoaf et al, 1998	Whittier Narrows 1987; Loma Prieta 1989; Northridge 1994; all in California, USA	Injury	1.6	1.0–2.5	1.7 (1.2–2.3)
	Peek-Asa et al, 2003	1994 Northridge, California	Injury	2.4	1.2–5.1	
	Doocy et al, 2009	2007 Ica, Peru	Injury	1.6	1–2.7	
Physical disability						
	Chou et al, 2004	1999 Chi-Chi, Taiwan	Death	1.7	1.2–2.3	1.8 (0.8–4)*
	Osaki & Minowa 2001	1995 Great Hanshin, Japan	Death	1.1	0.5–2.3	
Socioeconomic status						
	Chou et al, 2004	1999 Chi-Chi, Taiwan	Death	2.2	1.6–3	2.2 (1.6–3)*

*used both for injury and death equations due to lack of other relevant data.

#### Population data acquisition

The study was conducted in the city of Tiberias; located on the western coast of the Sea of Galilee and a major population center in Northern Israel with approximately 40,000 residents. Its location near the Dead Sea Fault (a tectonically active plate boundary) makes it a highly vulnerable area to earthquakes [24]. Data was obtained from the Israeli Central Bureau of Statistics regarding all twelve census tracts of the city. For all city residents, information was gathered regarding the four risk-factors mentioned above; the data was gathered dichotomously: ‘yes’ (1) = in the existence of a risk factor and ‘no’ (0) = in the absence of a risk factor. Thus, individuals were marked as ‘yes’ or ‘no’ for each risk factor: being a female; being over the age of 65; having a physical disability (defined as mobility impairment at all levels); and having low socioeconomic status (defined as having an annual income lower than the national average).

#### Model structure and settings

The integrative model was formulated based on a logistic regression equation that included both the assumptions of the HAZUS model and the four risk factors mentioned above:

$$p=\frac{1}{1+e^{-(\beta_{0}+\beta_{1}X_{1}+\beta_{2}X_{2}+\beta_{3}X_{3}+\beta_{4}X_{4})}}$$

*p =* the probability of an individual of suffering an injury or dying in an earthquake

β_0_—is the value provided by HAZUS (according to the level of structural damage)

β_1_ is the combined effect of age>65 on the risk of injury/death in earthquakes

β_2_—is the combined effect of gender (female) on the risk of injury/death in earthquakes

β_3_-is the combined effect of having a physical disability on the risk of injury/death in earthquakes

β_4_—is the combined effect of low socioeconomic status on the risk of injury/death in earthquakes

X_1-4_ –represents the presence of a risk factor, defined as 1 = ‘yes’ or 0 = ‘no’.

The value of β_0_ changes according to the level of structural damage and the compatible casualty rates in HAZUS (see Table 2). For example, when a structure collapses, the HAZUS model assumes that 10% (*p* = 0.10) of the occupants will be killed. This is used to calculate β_0_:

$$0.10=\frac{1}{1+e^{\beta_{0}}}\to \beta_{0}=-log(9)\to -2.197$$

**Table 2:** HAZUS casualty rates according to building^*^ damage and injury severity.

	Light injury (severity 1)	Moderate injury (severity2)	Severe injury (severity 3)	Fatal injury /death (severity 4)
Slight structural damage	0.05	0.005	0	0
Moderate structural damage	0.25	0.03	0	0
Severe structural damage	1	0.1	0.001	0.001
Complete structural damage–no collapse	5	1	0.01	0.01
Complete structural damage–with collapse	40	20	5	10

*The rates presented here are for reinforced concrete structures which are the common residential structures in Israel

This value is then entered into the equation along with the combined effects calculated from the meta-analysis procedure and the population data. For example, for estimating the probability of an occupant to die when a structure collapses the following equation was constructed:

$$p=\frac{1}{1+e^{-(-2.197+1.073X_{1}+0.182X_{2}+0.609X_{3}+0.788X_{4})}}$$

#### Casualty estimation procedure–the case of Tiberias

For the estimation procedure, a hypothetical (worst case) scenario was assumed in which all the structures in Tiberias suffered heavy damage and collapse, although in an actual event the degree of damage will most likely vary. The estimation procedure was performed for each census tract of the city (n = 12) separately.

The procedure included four steps comprising four equations according to injury levels. Since the combined effects calculated in the meta-analysis excluded a reference to injury levels, the same combined measures (apart from β_0_) were used to assess casualties in all three injury severity levels (i.e. slight, moderate and severe). Regarding two risk factors (having a physical disability and low socioeconomic status) no studies were found that reported effect size measures related to injury (only to death). In these cases, the combined effects that were calculated (related to the risk of death) were also used in the equations that estimated the expected number of injuries. To address these issues, the proposed model was characterized by a hierarchical structure, that resembles the approach used in multinomial logistic regression and is familiar in the field of road accidents and car occupants’ injury severity [25–26], based on the concept that a combination of risk factors leads to a more severe outcome [27] as do multiple injuries [28]. This led to individuals with higher injury or death probabilities (calculated in the model) to first be assigned into higher-severity casualty groups compared with those who had fewer risk factors and lower injury or death probabilities.

**Fig 1:**
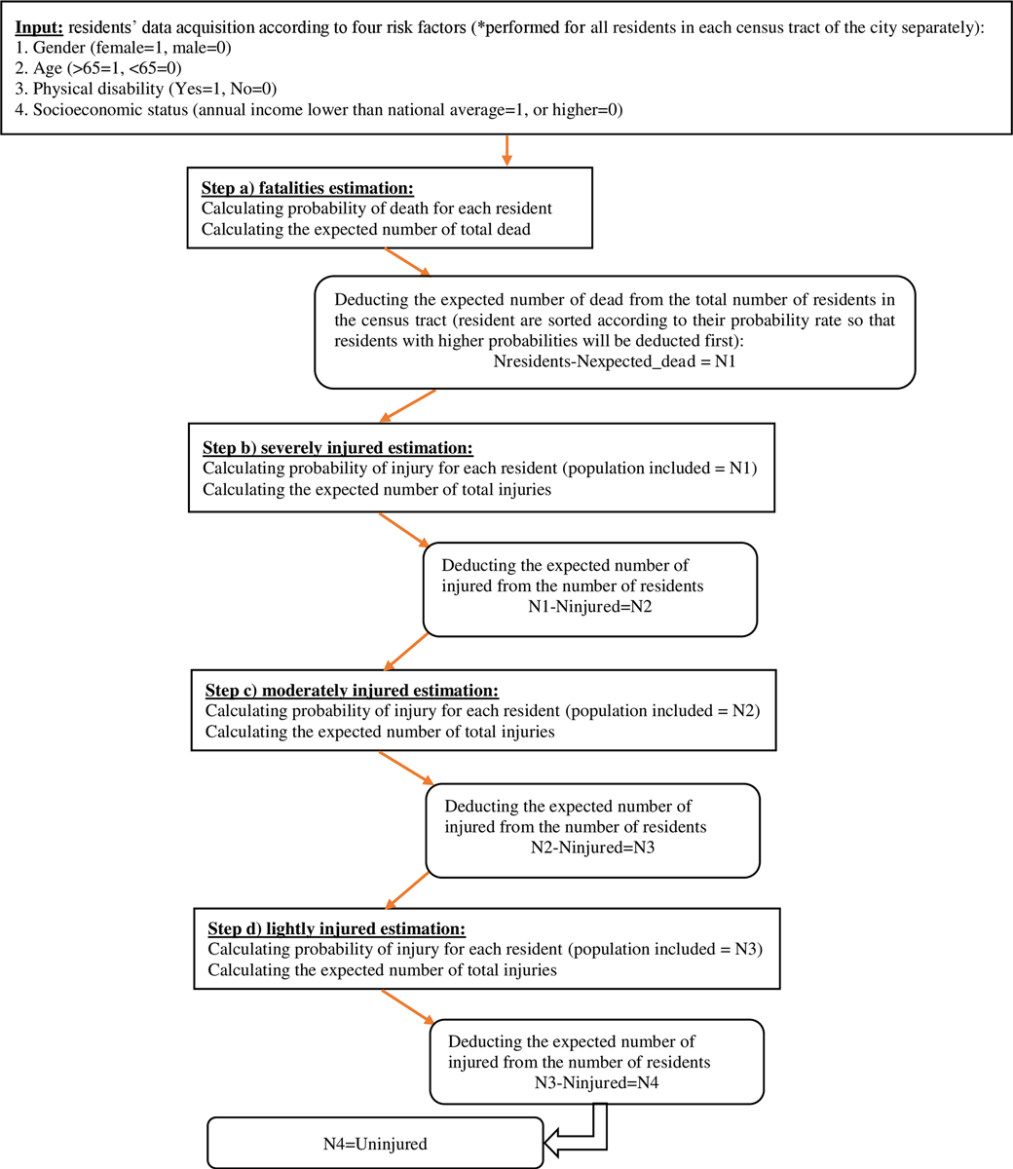
Flowchart of the casualty estimation procedure in the proposed integrated model

Step (a) estimation of expected fatalities: for each resident the probability of dying when a structure collapse was calculated according to the equation detailed above. The expectation E[X] was calculated (the sum of all probabilities) in order to determine the number of fatalities; this number was subtracted from the total number of residents in the census tract (residents with the highest death probabilities were excluded first and so on) and the reminder was then transferred to serve as the basis for step (b)–estimation of expected severely injured, which was performed in the same method as in step (a). Steps (c) and (d) estimated the moderate and slight injuries, again calculated in the same manner. After ending the procedure (following step (d)), the remainder of the residents were defined as uninjured. The entire estimation procedure is detailed in Fig 1.

## Results

### Meta-analysis of risk factors related to injury and death in earthquakes

Age was the strongest factor increasing the risk of death in earthquakes, followed by socioeconomic status, physical disability and gender. Individuals aged 65 years and older had the highest combined OR for increased risk of dying in an earthquake of 2.92 (95% CI 0.99, 8.6) compared to younger individuals. Two studies directly assessed the impact of age and gender with regard to the risk of injury; the results indicated that gender has a higher combined OR of 1.7 (95% CI 1.2, 2.3) compared with age (OR = 1.3, 95% CI 0.3, 5.3). No studies were found that measured the direct impact of socioeconomic status and physical disability on the risk of injury and therefore, the combined effect that was calculated for the risk of death was used for injury as well. The combined effect measures are detailed in Table 1.

### Population characteristics–the city of Tiberias

The data gathered regarding the rate of risk factors related to individual and household characteristics in the population revealed disparities in different census tracts (neighborhoods) in Tiberias. The difference in the rate of residents older than 65 years in the various census tracts of the city ranged between 1.5%-16.5%. The rate of physically disabled persons ranged between 3.5%-27.5% in different census tracts. Gender distribution was similar among all census tracts (mean value = 49.8%). The average annual income of the census tracts residents was compared with the national average and found to be lower than the national average among 10 out of 12 census tracts.

### Casualty estimation in the city of Tiberias using the integrated model

The results of the casualty estimation procedure according to twelve census tracts of the city of Tiberias are detailed in Table 3. The results indicate that the number of fatalities in this given scenario more than doubled when integrating individual and household characteristics to the estimation procedure (24% in the integrated model vs. 10% in HAZUS traditional model). Similar results are shown for severely injured casualties (9% vs. 5%). A smaller increase of 5% is observed for moderately injured casualties (25% vs. 20%) whereas the rate of slightly injured casualties is decreased by almost half compared with the results of the HAZUS traditional model (23% vs. 40% respectively).

**Table 3:** Results of casualty estimation simulation–integrated model (according to census tracts).

Census tract*	Number of residents	Fatal Injury	%	Severe Injury	%	Moderate injury	%	Light Injury	%	No injury	%	data on census tract characteristics				
												SEI rank	% >65	Gender—female (%)	% of physically disabled	above average salary
11	5926	1487	25	583	10	1547	26	1373	23	936	16	10	12	50.5	9	yes
12+13	7023	1602	23	655	9	1773	25	1655	24	1339	19	7,8	8	50	8	no
14	1205	313	26	114	9	305	25	280	23	193	16	7	15	48	11	no
15	1198	334	28	112	9	297	25	261	22	192	16	7	15	46	27	no
21	5122	1209	24	471	9	1271	25	1168	23	1003	20	9	11	50	13.5	no
22+23	4638	1129	24	439	9	1167	25	1033	22	870	19	8,9	12.5	54	11	no
24	2152	426	20	114	5	581	27	538	25	491	23	9	1.5	52	3.5	yes
25	3038	715	24	279	9	754	25	698	23	592	19	9	8	50	17	no
31+32	5467	1346	25	511	9	1372	25	1249	23	989	18	6	10	50	17.5	no
33	1752	482	28	169	10	396	23	418	24	287	16	6	14	50	23	no
34	1974	461	23	194	10	517	26	464	24	338	17	6	5	48	14	no
36	2584	610	24	200	8	692	27	610	24	469	18	6	16.5	49	27.5	no
**Average injury rate for each injury category**																
Current model			24		9		25		23		18					
HAZUS rates			10		5		20		40		25					

*In order to resolve some discrepancies concerning the city’s area partition by the census (of the Israeli bureau of statistics) and the building stock catalog in HAZUS, some census tracts were grouped together in pairs.

When examining the casualty rates among various census tracts, it becomes clear that certain areas bear a heavier casualty burden compared to others. Areas with relatively low rates of vulnerable populations (manifested in the risk factors defined previously) are likely to have almost 10% less fatalities than more vulnerable areas in this regard; for example census tract 24 is expected to have 20% fatalities versus 28% in census tract 33 that include much higher rates of elderly population (over 65) and physically disabled population and are also ranked lower in the socioeconomic index. These differences are also demonstrated regarding severely injured casualties (5% expected in census tract 24 vs. 10% in census tract 33). When examining the predicted values of moderately and slightly injured casualties the differences seem to decrease; the difference between the predicted lowest value and the highest value of moderately injured casualties is 4% and 2% for slightly injured casualties.

## Discussion

This paper provides an innovative integrated and interdisciplinary practical approach to estimate the number of earthquake-induced casualties of different severity levels using a combination of human-related and structural factors. The results of the new model were compared with a traditional engineering-based model. The integration of human-related risk factors altered both the general expected number of casualties in a given event and more importantly, the composition of casualties (percentage of casualties in each severity category). There was an increase in the overall number of casualties and in the expected number of fatalities, severe and moderate injuries, and a decrease in the expected number of slight injuries. Geographical variability in vulnerability was also demonstrated, as areas of Tiberias with higher rates of at-risk population were identified to be more vulnerable in this regard. When interpreting these results, one should note that since the examined area’s social and demographic attributes has a great impact on the outcomes of the model, two sub-populations located within a small geographical distance may produce different results in the same earthquake simulation. In the current study, the city of Tiberias, is ranked relatively low in the socioeconomic index compared to other municipalities in the country [24]. Since this is one of the risk factors taken in account in the model, it increases the probabilities of injury and death of its residents even before the interaction with other risk factors and regardless of the physical damage to structures. The premise, basically underlying the model, that the social vulnerability of a community is highly correlated with the potential number of casualties from natural disasters is demonstrated in several types of events, not only earthquakes. The hurricanes of 2005 in the gulf coast of the United States, provided strong evidence that social disparities, derived from race, socioeconomic status, gender, and age of the affected communities have resulted in an uneven impact of the calamity [29–31]; similar findings were manifested in climatological disasters, where certain neighborhoods of a US metropolitan area were more vulnerable in terms of heat-related deaths in relation to the residents income level and age [32].

The results of the current model along with the evidence from the literature may indicate a potential gap in the estimation process of earthquake-related casualties resulting from the previously missing human characteristics dimension. This conjecture is also supported by other evidence dealing with the level of uncertainty of the traditional HAZUS model evaluated in several validation assessments that manifested among others in inaccuracies of the casualty estimates [33–36]. Further research may establish this issue. Another possible explanation for the gaps in projections is that the experts who formulated the casualty rates for the HAZUS model based it on an average case mix of population characteristics (e.g. different levels of socioeconomic status; age composition etc.); therefore, integrating the data obtained in this study may have resulted in an overestimation of the number of casualties. However, this issue is not discussed clearly in the literature related to the HAZUS methodology, and furthermore, adding risks to different strata of population may increase the model sensitivity. In addition, an overestimation of casualties is preferable to an underestimation that might lead to a decrease in first response capabilities.

The addition of human-related risk factors to the estimation process of earthquake-related casualties is part of a more comprehensive approach of risk assessment [5,37], and may have an added value in other areas as well. The process itself provides valuable data that could also be used for creating “social risk maps” indicating risk prone areas or neighborhoods. A possible use of this data could be forming contingency plans dealing with special needs of vulnerable population prior and post a disaster (e.g. the delivery of medications to elderly population in order to avoid post-disaster comorbidity) [9,38].

Several limitations of the current study derive from the scarcity of data regarding the effect size measures of human-related risk factors. Other result from difficulties in obtaining reliable data from areas steeped in chaos [39]. Further research is needed to strengthen existing data and provide accurate and detailed information in this regard, designed to improve casualty estimation. Nevertheless, the method offered by this paper for integrating epidemiological data to engineering-based models can be calibrated at any point when new and updated studies will be published. Another limitation arises as validation of the model estimates compared to real event results is not possible since as mentioned, the last deadly earthquake is Israel occurred decades ago and proper documentation of casualty number is not available. Another issue is that earthquake vulnerability in regards to risk factors related to the exposed population may vary depending on the examined area, but again the methodology offered here is flexible and can be modified in accordance with the information available to potential users.

The implications and use of this novel approach are wide ranging. Previously, casualty estimation was based solely on engineering methods and damage to the build environment. As suggested by this paper, the expected number of casualties can be estimated utilizing a more comprehensive approach which incorporates social vulnerability of the investigated area.

## Conclusion

This study demonstrates the use of an innovative approach which takes into account both the built environment and population characteristics to predict earthquake casualties. Such knowledge may lead to more focused investment of efforts in reducing vulnerability of potentially more severely affected populations, prior to an occurrence of an earthquake.

## Supporting Information

S1 Table Details of the equations building process. (XLSX)
